# Coenzyme Q10 ameliorates oxidative stress and prevents mitochondrial alteration in ischemic retinal injury

**DOI:** 10.1007/s10495-013-0956-x

**Published:** 2013-12-12

**Authors:** Dongwook Lee, Keun-Young Kim, Myoung Sup Shim, Sang Yeop Kim, Mark H. Ellisman, Robert N. Weinreb, Won-Kyu Ju

**Affiliations:** 1 Laboratory for Optic Nerve Biology, Department of Ophthalmology, Hamilton Glaucoma Center, University of California San Diego, 9415 Campus Point Drive, La Jolla, CA 92037 USA; 2 Research Institute of Clinical Medicine of Chonbuk National University-Biomedical Research Institute, Chonbuk National University Hospital, Chonju, Chonbuk Republic of Korea; 3 Department of Neuroscience, Center for Research on Biological Systems, National Center for Microscopy and Imaging Research, University of California San Diego, La Jolla, CA USA

**Keywords:** Coenzyme Q10, Retinal ischemia, Retinal ganglion cell, Oxidative stress, Mitochondrial transcription factor A, Mitochondrial DNA

## Abstract

Coenzyme Q10 (CoQ_10_) acts by scavenging reactive oxygen species for protecting neuronal cells against oxidative stress in neurodegenerative diseases. We tested whether a diet supplemented with CoQ_10_ ameliorates oxidative stress and mitochondrial alteration, as well as promotes retinal ganglion cell (RGC) survival in ischemic retina induced by intraocular pressure elevation. A CoQ_10_ significantly promoted RGC survival at 2 weeks after ischemia. Superoxide dismutase 2 (SOD2) and heme oxygenase-1 (HO-1) expression were significantly increased at 12 h after ischemic injury. In contrast, the CoQ_10_ significantly prevented the upregulation of SOD2 and HO-1 protein expression in ischemic retina. In addition, the CoQ_10_ significantly blocked activation of astroglial and microglial cells in ischemic retina. Interestingly, the CoQ_10_ blocked apoptosis by decreasing caspase-3 protein expression in ischemic retina. Bax and phosphorylated Bad (pBad) protein expression were significantly increased in ischemic retina at 12 h. Interestingly, while CoQ_10_ significantly decreased Bax protein expression in ischemic retina, CoQ_10_ showed greater increase of pBad protein expression. Of interest, ischemic injury significantly increased mitochondrial transcription factor A (Tfam) protein expression in the retina at 12 h, however, CoQ_10_ significantly preserved Tfam protein expression in ischemic retina. Interestingly, there were no differences in mitochondrial DNA content among control- or CoQ_10_-treated groups. Our findings demonstrate that CoQ_10_ protects RGCs against oxidative stress by modulating the Bax/Bad-mediated mitochondrial apoptotic pathway as well as prevents mitochondrial alteration by preserving Tfam protein expression in ischemic retina. Our results suggest that CoQ_10_ may provide neuroprotection against oxidative stress-mediated mitochondrial alterations in ischemic retinal injury.

**Electronic supplementary material:**

The online version of this article (doi:10.1007/s10495-013-0956-x) contains supplementary material, which is available to authorized users.

## Introduction

Elevated intraocular pressure (IOP) is an important risk factor for retinal ganglion cell (RGC) death and optic nerve degeneration in retinal ischemia and glaucoma [1]. Growing evidence indicates that ischemic injury by acute IOP elevation alters mitochondrial dynamics-related proteins and induces mitochondrial dysfunction-mediated apoptotic cell death in the retina of rodent models [2, 3]. Importantly, our previous studies demonstrated that IOP elevation triggered release of mitochondrial fusion protein optic atrophy 1 and increase of mitochondrial fission GTPase dynamin related protein-1, as well as induced apoptotic cell death by modulating Bax/phosphorylated Bad (pBad) protein expression in ischemic retina [2, 3]. Collectively, these findings suggest that ischemic injury induced by acute IOP elevation is associated with mitochondrial dysfunction-mediated apoptotic pathway in the retina.

Oxidative stress has been implicated as an important pathophysiological mechanism for mitochondrial dysfunction-mediated retinal neurodegeneration including ischemic injury [4–7]. Superoxide dismutases (SODs), cytosolic SOD1 and mitochondrial SOD2, are expressed in the ganglion cell layer (GCL) and inner plexiform layer in rodent retina [8]. A recent study suggests that SOD2 has a protective role against oxidative stress-mediated neuronal cell death [9]. Of interest, we found that acute IOP elevation significantly increased SOD2 protein expression in the early neurodegeneration of ischemic rat retina [5]. In addition, increasing evidence indicates that oxidative stress is associated with mitochondrial DNA (mtDNA) alteration-related mitochondrial dysfunction in retinal neurodegeneration [5, 7, 10, 11]. However, it remains unknown whether oxidative stress induced by acute IOP elevation causes mtDNA alteration in ischemic retinal injury.

Mitochondrial transcription factor A (Tfam, also as known as mtTFA), a nucleus encoded DNA-binding protein in mitochondria, has an important role in mitochondrial gene expression and mtDNA maintenance, and therefore is essential for oxidative phosphorylation (OXPHOS)-mediated adenosine triphosphate (ATP) synthesis [12–15]. Mice lacking *Tfam* have impaired mtDNA transcription and loss of mtDNA leads to bioenergetics dysfunction and embryonic lethality [12]. In contrast, overexpression of Tfam mediates delayed neuronal death following transient forebrain ischemia in mice [16–18] as well as neonatal hypoxic-ischemic brain injury rapidly increased Tfam and OXPHOS complex IV protein expression in a rat model [19]. This suggests that these responses may support endogenous repair mechanisms for mtDNA damage following hypoxic-ischemic brain injury [19]. Of note, acute IOP elevation significantly increased Tfam and OXPHOS complex protein expression in the early neurodegeneration of ischemic rat retina [5], collectively suggesting that these responses may reflect endogenous repair mechanisms for elevated IOP-induced mitochondrial alteration in ischemic injury.

Coenzyme Q10 (CoQ_10_), an essential cofactor of the electron transport chain, acts by maintaining the mitochondrial membrane potential, supporting ATP synthesis and inhibiting reactive oxygen species (ROS) generation for protecting neuronal cells against oxidative stress in neurodegenerative diseases [20–22]. Previous studies have demonstrated that CoQ_10_ protected retinal neurons against hydrogen peroxide-induced oxidative stress in vitro [23] as well as prevented retinal damage caused by acute high IOP-induced transient ischemic injury [24, 25].

In the current study, we tested whether a diet supplemented with CoQ_10_ ameliorates oxidative stress-mediated apoptotic cell death in RGC degeneration by preventing mitochondrial alteration in ischemic mouse retina.

## Materials and methods

### Animals

Female, 4-month-old C57BL/6 mice (20–25 g in weight; The Jackson Laboratories, Bar Harbor, ME, USA) were housed in covered cages, fed with a standard rodent diet ad libitum, and kept on a 12 h light/12 h dark cycle. All procedures concerning animals were performed in accordance with the ARVO statement for the Use of Animals in Ophthalmic and Vision Research and under protocols approved by institutional IACUC committees at the University of California San Diego.

### Induction of transient retinal ischemia

The mice were anesthetized with a mixture of ketamine (100 mg/kg, Ketaset; Fort Dodge Animal Health, Fort Dodge, IA, USA) and xylazine (9 mg/kg, TranquiVed; Vedeco, Inc., St. Joseph, MO, USA) by intraperitoneal (IP) injection. Eyes were also treated with 1 % proparacaine drops. A 30-gauge needle was inserted into the anterior chamber of right eye that was connected by flexible tubing to a saline reservoir. By raising the reservoir, IOP was elevated to 70–80 mmHg for 50 min. Sham treatment was performed in the contralateral eyes by the insertion of a needle in the anterior chamber without saline injection. Retinal ischemia was confirmed by observing whitening of the iris and loss of the retina red reflex. IOP was measured with a tonometer (TonoLab; Tiolatoy, Helsinki, Finland) during ischemia. Non-ischemic contralateral control retinas were used as control.

### Pharmacological treatment

CoQ_10_ was purchased from Kaneka Nutrients (Pasadena, TX, USA) or Sigma (St. Louis, MO, USA). AIN-93G purified control or a diet supplemented with CoQ_10_ were formulated by Harlan Laboratories (Madison, WI, USA). Four groups of mice were studied: a group of non-ischemic C57BL/6 mice treated with control diet (*n* = 20 mice), a group of ischemic C57BL/6 mice treated with control diet (*n* = 30 mice), a group of non-ischemic C57BL/6 mice treated with 1 % CoQ_10_ diet [(v/v), which equals a daily dose of 1,600–2,000 mg/kg body weight in 25–30 g mice, *n* = 20 mice] [26] and a group of ischemic C57BL/6 mice treated with 1 % CoQ_10_ diet (*n* = 30 mice).

### Tissue preparation

Six to 24 h after acute IOP elevation, light adapted mice were anesthetized with IP injection of mixture of ketamine/xylazine, as described, and then mice were perfused transcardially with 0.9 % saline followed by 4 % paraformaldehyde in 1X phosphate buffer saline (PBS, pH 7.4). Both eyes enucleated and fixed in 4 % paraformaldehyde in PBS for 4 h at 4 °C. After several washes in PBS, the retinas were dissected and then dehydrated through graded ethanol solutions and embedded in polyester wax, as described previously [27].

### Whole-mount immunohistochemical analysis

Retinas from enucleated eyes were dissected as flattened whole-mounts at 12 h or 2 weeks after ischemia–reperfusion. Retinas were immersed in PBS containing 30 % sucrose for 24 h at 4 °C. The retinas were blocked in PBS containing 3 % donkey serum, 1 % bovine serum albumin, 1 % fish gel and 0.1 % Triton X-100 for 1 h, and incubated with goat polyclonal anti-Brn3a antibody (1:500; Santa Cruz Biotechnology, Santa Cruz, CA, USA), a specific maker for RGCs [28, 29], and guinea pig polyclonal anti-GFAP antibody (1:500; Advanced ImmunoChemical, Long Beach, CA, USA) for 3 days at 4 °C. After several wash steps, the retinas were incubated with the secondary antibodies, Alexa Fluor-568 donkey anti-goat IgG antibody or Cy5-conjugated anti-guinea pig IgG antibody (Invitrogen, Carlsbad, CA, USA) for 24 h, and subsequently washed with PBS. The retinas were counterstained with the nucleic acid stain Hoechst 33342 (1 μg/mL; Invitrogen) in PBS. Images were captured under fluorescence microscopy (Eclipse microscope, model E800; Nikon instruments Inc., Meliville, NY, USA) equipped with a digital camera (SPOT; Diagnostic Instrument, Sterling Heights, MI, USA). Images were acquired with commercial software (Simple PCI version 6.0 software; Compix Inc., Cranberry Township, PA, USA).

### Quantitative analysis for RGC counting

To count RGCs labeled with Brn3a, each retinal quadrant was divided into three zones by central, middle, and peripheral retina [one sixth (~400 μm), three sixths (~1,200 μm), and five sixths (~2,000 μm) of the retinal radius from the optic nerve head]. Images were taken at 20×, covering an area of 0.344 mm^2^, and then the number of RGCs were normalized per mm^2^. RGC densities were measured in 24 distinct areas (two areas at central, middle, and peripheral per retinal quadrant) per condition by two investigators in a masked fashion, and the scores were averaged (*n* = 7 retinas/group). To further examine RGC survival between control and CoQ_10_ diet-treated non-ischemic retinas, RGC densities were automatically measured using ImageJ cell counting analysis (http://rsb.info.nih.gov/ij/, National Institute of Health, Bethesda, MD, USA).

### Immunohistochemical analysis

Immunohistochemical staining of retinal cross sections at 12 h after transient ischemia was performed as previously described [27]. Five sections per wax block from each group were used for immunohistochemical analysis. Primary antibodies were goat polyclonal anti-Brn3a antibody (1:500; Santa Cruz Biotechnology, Santa Cruz, CA, USA), a specific maker for RGCs [30], monoclonal mouse anti-GFAP antibody (1:500; Sigma, St. Louis, MO, USA) and rabbit polyclonal anti-Iba1 antibody (1:500; Wako Chemicals USA, Inc., Richmond, VA, USA). To prevent non-specific background, tissues were incubated in 1 % bovine serum albumin/PBS for 1 h at room temperature before incubation with the primary antibodies for 16 h at 4 °C. After several wash steps, the tissues were incubated with the secondary antibodies, Alexa Fluor 568 dye-conjugated donkey anti-goat IgG antibody (Invitrogen, Carlsbad, CA, USA), Cy5 dye-conjugated goat anti-mouse IgG antibody (Invitrogen) or Alexa Fluor 488 dye-conjugated anti-rabbit IgG antibody (1:100; Invitrogen) for 4 h at 4 °C and subsequently washed with PBS. The sections were counterstained with the nucleic acid stain Hoechst 33342 (Invitrogen) in PBS. Images were acquired with confocal microscopy (Olympus FluoView1000; Olympus, Tokyo, Japan).

### Western blot analysis

Following 6, 12 or 24 h after transient ischemia, the collected retinas were homogenized in a glass-Teflon Potter homogenizer in RIPA lysis buffer (150 mM NaCl, 1 mM EDTA, 1 % NP-40, 0.1 % SDS, 1 mM DTT, 0.5 % sodium deoxycholate and 50 mM Tris-Cl, pH 7.6) containing complete protease inhibitors (Roche Biochemicals, Indianapolis, IN, USA). Each sample (10 μg; *n* = 3 retinas/group) was separated by PAGE and electrotransferred to polyvinylidenedifluoride membrane. The membrane was blocked with 5 % nonfat dry milk and 0.1 % Tween-20 in PBS for 1 h, incubated with rabbit polyclonal anti-SOD2 antibody (1:5000; Santa Cruz Biotechnology), rabbit polyclonal anti-HO-1 antibody (1:1000; Millipore), monoclonal mouse anti-GFAP (1:3000; Sigma), rabbit polyclonal anti-Iba-1 antibody (1:2000; Wako Chemical USA), mouse monoclonal anti-caspase antibody (1:3000; Cell Signaling, Danvers, MA, USA), rabbit polyclonal anti-Bax antibody (1:500; Santa Cruz Biotechnology), mouse monoclonal anti-phosphorylated Bad (pBad, 1:2000; Cell Signaling), goat polyclonal anti-Tfam antibody (1:1000; Santa Cruz Biotechnology), rabbit polyclonal anti-Porin (1:1000; Calbiochem, La Jolla, CA, USA) and mouse monoclonal anti-actin antibody (1:10,000, Millipore, Billerics, MA, USA) for overnight at 4 °C. After several washes in Tween/PBS, the membranes were incubated with peroxidase-conjugated donkey anti-goat IgG (1:5000: Bio-Rad, Hercules, CA, USA), goat anti-rabbit IgG (1:5000; Bio-Rad) or goat anti-mouse IgG (1:5000; Bio-Rad), and developed using chemiluminescence detection (ECL Plus; GE Healthcare Bio-Science, Piscataway, NJ, USA). The scanned film Images were analysed by ImageJ (National Institute of Health) and band densities were normalized to the band densities for actin or porin.

### TUNEL staining

The retinal cross sections at 12 h after transient ischemia were incubated with proteinase K (10 μg/mL, 10 mM Tris, pH 7.4–8.0) for 10 min at 37 °C. After rinsing in PBS, the sections were incubated with terminal deoxynucleotidyl transferase plus nucleotide mixture in reaction buffer for 60 min at 37 °C (In situ Cell Death Detection kit, Roche Applied Science, Indianapolis, IN, USA) as previously described [3]. To count TUNEL-positive cells, the areas were divided into three layers by GCL, inner nuclear layer (INL) and outer nuclear layer (ONL). TUNEL-positive cells were counted in eight microscopic fields of 0.2 mm from retinal sections per condition (*n* = 5 retinas/group) by two investigators in a masked fashion, and the scores were averaged.

### Measurement of mtDNA content

MtDNA content of each sample at 12 h after transient ischemia was determined as described previously [31]. Briefly, total genomic DNA (gDNA) was isolated from retinas by using DNeasy^®^ Blood & Tissue Kit (Qiagen, Valencia, CA, USA) as described in the manufacturer’s protocol. For the measurement of relative mtDNA content, real-time PCR was carried out using MX3000P real-time PCR system (Stratagene, La Jolla, CA, USA) as following. Total gDNAs (10 ng) from the each sample were amplified using iQTM SYBR^®^ Green supermix (Bio-Rad, Hercules, CA, USA) and mitochondrial cytocrome B (CytB-F: 5′-GGTCTTTTCTTAGCCATACACTACA-3′; CytB-R: 5′-ATATCGGATTAGTCACCCGTAAT-3′) or β-actin (ActB-F; GATCGATGCCGGTGCTAAGA-3′; ActB-R: 5′-CACCATCACACCCTGTGGAAG-3′) primers for 40 cycles [initial incubation at 95 °C for 10 min, and 40 cycles (95 °C for 30 s, 55 °C for 30 s and 72 °C for 20 s)]. Output data were obtained as Ct values and the difference of mtDNA content among samples was calculated using the comparative Ct method [32]. ActB gene was used to normalize the ratio between mtDNA and gDNA. The samples were run in triplicates for all experiment.

### Statistical analysis

Data were presented as the mean ± SD. Comparison of two or three experimental conditions was evaluated using the unpaired, two-tailed Student’s *t* test or one-way analysis of variance and the Bonferroni *t* test. *P* < 0.05 was considered to be statistically significant.

## Results

### The effect of CoQ_10_ in IOP and body weight

**Fig. 1:**
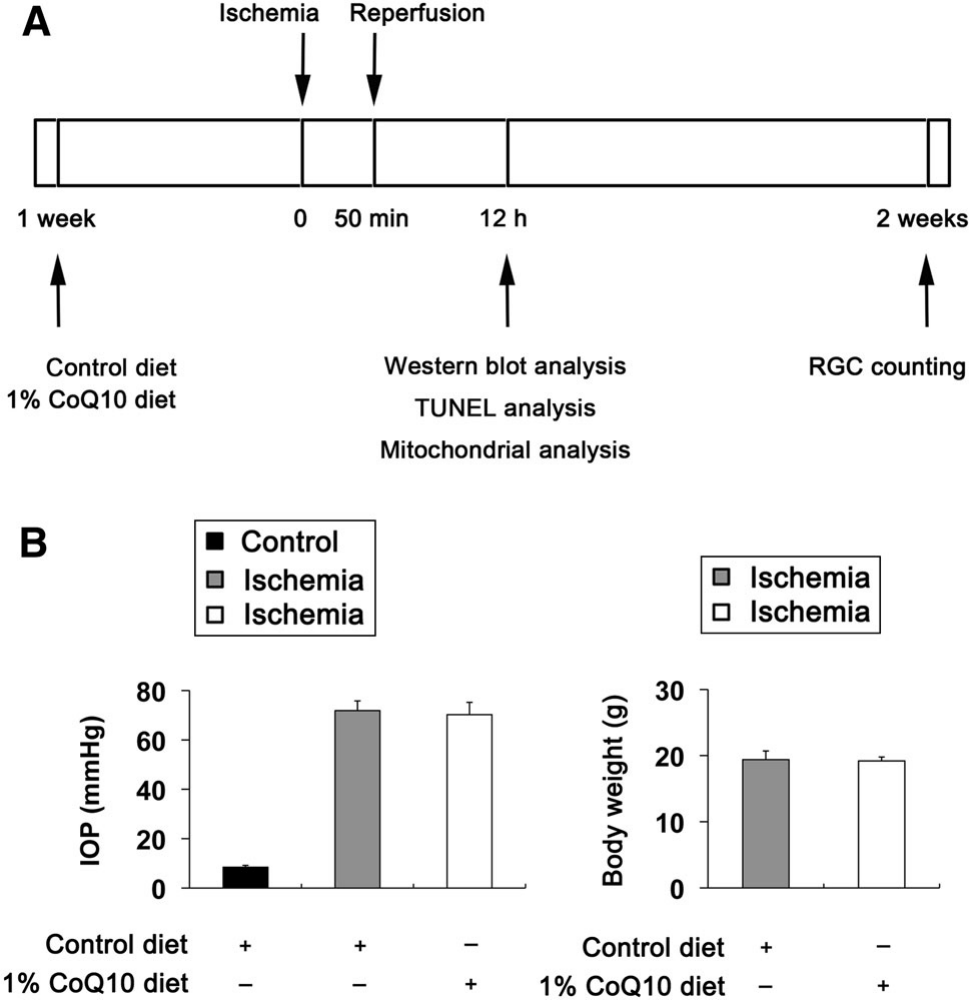
CoQ_10_ supplementation and induction of transient retinal ischemia. **a** Diagram for control and CoQ_10_ (1 %) supplementation before and after ischemic injury. Unsupplemented control or CoQ_10_ diet were daily treated for 1 week before the induction of transient retinal ischemia and continued for 2 weeks. **b** IOP elevation in the mouse eyes and body weight in control diet- and CoQ_10_ diet-treated mice following transient retinal ischemic injury

We began either unsupplemented control or CoQ_10_ (1 %)-supplemented diet treatment daily for 1 week before the induction of transient retinal ischemia and then continued diet treatment for 2 weeks (Fig. 1a). Transient retinal ischemia was induced by acute IOP elevation to 71.8 ± 4.0 mmHg in mice treated with control diet (*n* = 30 mice) and 70.3 ± 5.0 mmHg in mice treated with CoQ_10_ diet (*n* = 30 mice) for 50 min (Fig. 1b). The pressure was enough to induce retinal ischemia and the phenotype was similar to pathologic acute angle closure glaucoma because when IOP reached 60 mmHg, the retina flow rate decreased by 68 % for retinal artery in rodent model [5, 33, 34]. The mean IOP of contralateral control eyes was 8.4 ± 0.7 mmHg (*n* = 20 mice; Fig. 1b). In addition, no difference was found in body weight between control and CoQ_10_ diet-treated mice during experimental period (*n* = 20 or 30 mice; Fig. 1b).

### CoQ_10_ ameliorates oxidative stress in ischemic retina

**Fig. 2:**
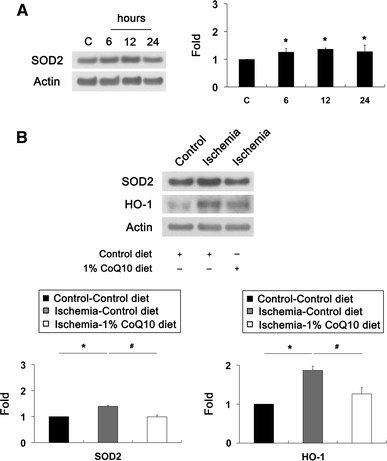
CoQ_10_-mediated blockade of oxidative stress in ischemic injury. Unsupplemented control or CoQ_10_ (1 %) diet were daily treated for 1 week before the induction of transient retinal ischemia and continued for 6, 12 or 24 h. **a** SOD2 Western blot analyses at 6, 12 and 24 h after transient retinal ischemia. Increase of SOD2 protein expression was maximal at 12 h in ischemic retina. The relative expression level of SOD2 protein was less at 24 h than at 12 h after ischemia–reperfusion. **b** SOD2 and HO-1 Western blot analyses at 12 h after transient retinal ischemia. CoQ_10_ treatment preserved SOD2 and HO-1 protein expression in ischemic retina compared with control diet-treated ischemic retina. Values are mean ± SD (*n* = 3 retinas/group). *Significant at *P* < 0.05 compared with control-diet-treated non-ischemic contralateral control retina or ^#^Significant at *P* < 0.05 compared with control diet-treated ischemic retina. *SOD2* superoxide dismutase 2, *HO*-*1* hemeoxygenase-1

We determined whether CoQ_10_ treatment prevents oxidative stress-mediated upregulation of superoxide dismutase 2 (SOD2) and heme oxygenase-1 (HO-1) protein expression using antibodies raised against SOD2 and HO-1. We observed that Increase of SOD2 protein expression was maximal 12 h later by 1.36 ± 0.04-fold in ischemic retina (*P* < 0.05; Fig. 2a). The relative quantity of SOD2 protein expression was less at 24 h than at 12 h after ischemia–reperfusion. Intriguingly, CoQ_10_ treatment preserved SOD2 and HO-1 protein expression in ischemic retina at 12 h compared with control diet-treated ischemic retina (*P* < 0.05; Fig. 2b), indicating that CoQ_10_ ameliorates oxidative stress in ischemic retinal injury.

### CoQ_10_ promotes RGC survival in ischemic retina

**Fig. 3:**
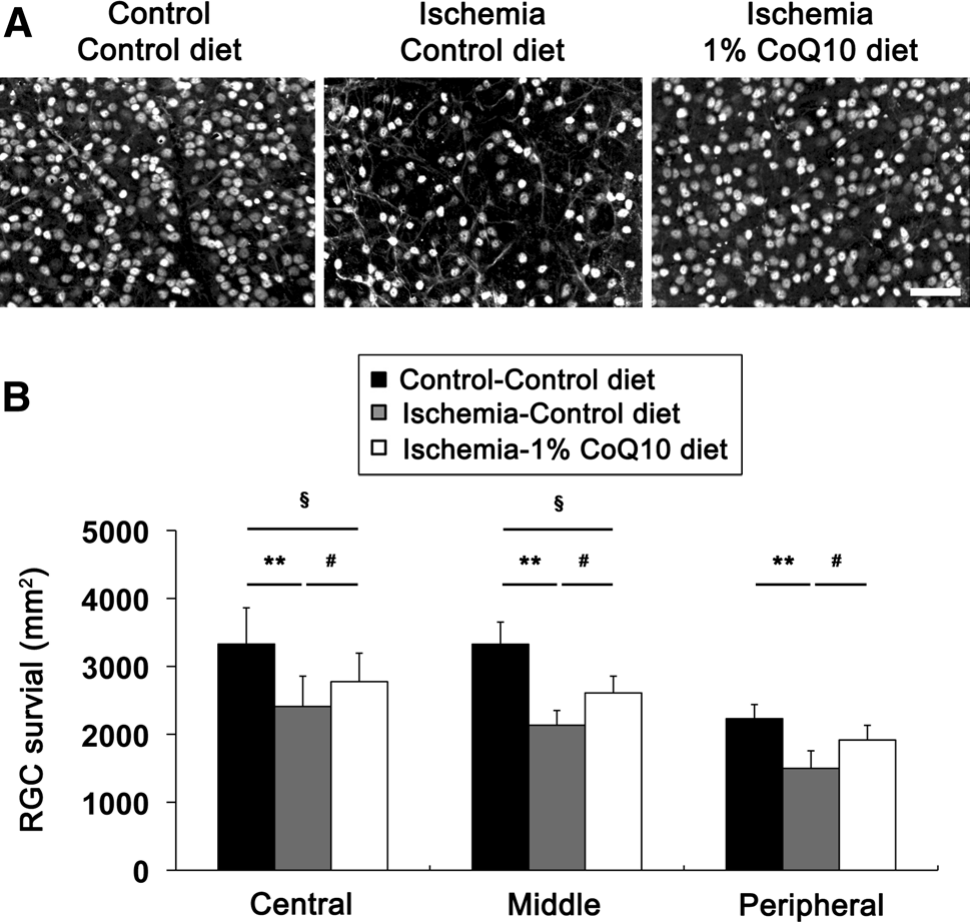
CoQ_10_-mediated protection of RGC survival in ischemic retina. Unsupplemented control or CoQ_10_ (1 %) diet were daily treated for 1 week before the induction of transient retinal ischemia and continued for 2 weeks. **a** Brn3a whole-mount immunohistochemistry at 2 weeks after transient retinal ischemia. High magnification showed representative images from the middle area of retinas. **b** Quantitative analysis of RGC survival. Values are mean ± SD (*n* = 7 retinas/group). ^*§*^Significant at *P* < 0.05 and **Significant at *P* < 0.01 compared with control diet-treated non-ischemic control retina or ^#^Significant at *P* < 0.05 compared with control diet-treated ischemic retina. *Scale bar* 50 μm

**Fig. 4:**
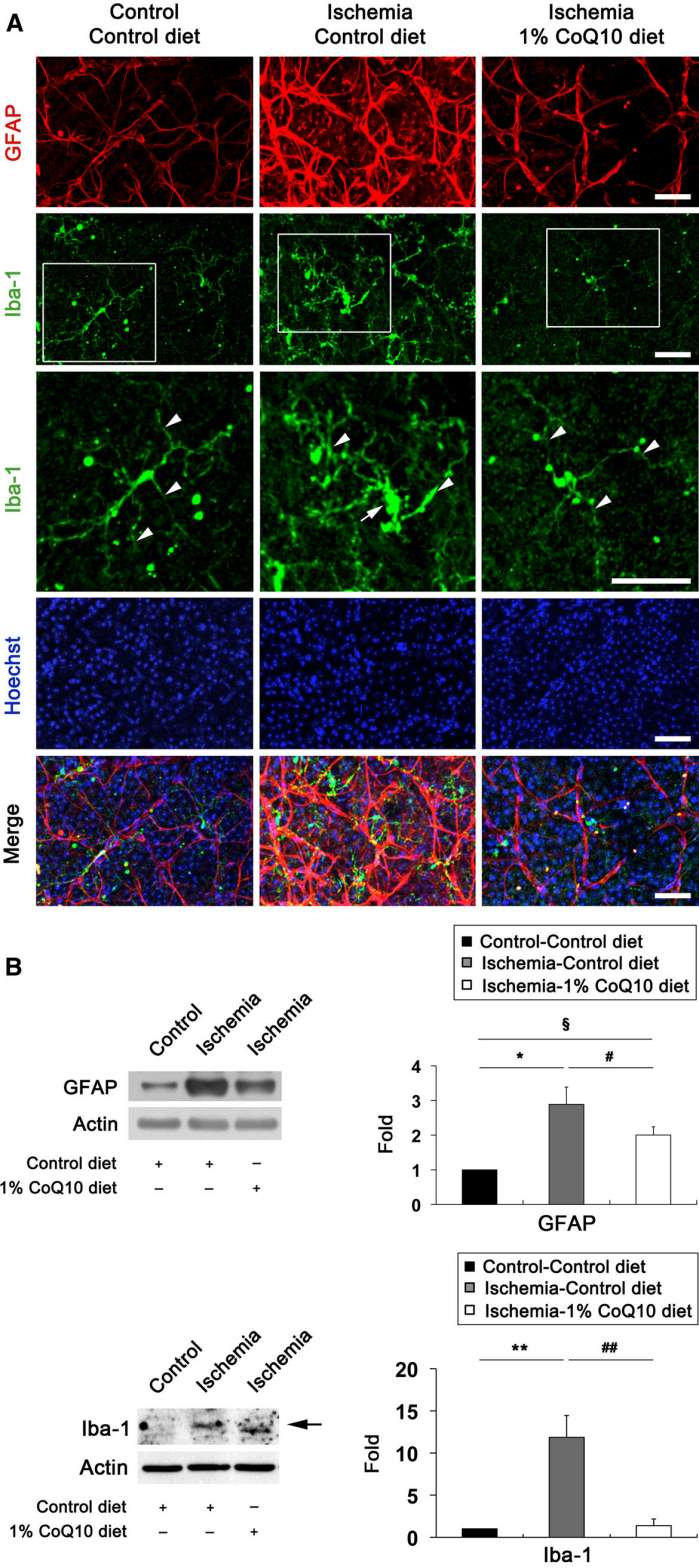
CoQ_10_-mediated blockade of astroglial and microglial activation in ischemic retina. Unsupplemented control or CoQ_10_ (1 %) diet were daily treated for 1 week before the induction of transient retinal ischemia and continued for 12 h. **a** Double immunohistochemistry for GFAP and Iba-1 at 12 h after transient retinal ischemia. Control diet-treated Ischemic retina increased GFAP and Iba-1 immunoreactivites in the GCL compared with control diet-treated non-ischemic control retina. In contrast, CoQ_10_ treatment decreased GFAP and Iba1 immunoreactivities in the GCL of ischemic retina. Intriguingly, higher magnification showed a quiescent microglial cell that has ramified long and thin processes in control diet-treated non-ischemic control retina and CoQ_10_ diet-treated ischemic retina (*arrowheads*). However, control diet-treated ischemic retina showed an activated microglial cell that has thickened processes (*arrowheads*) and swollen cell body (*arrow*). GCL ganglion cell layer. *Scale bars* 50 μm. **b** GFAP and Iba-1 Western blot analyses at 12 h after transient retinal ischemia. Control diet-treated ischemic retina significantly increased GFAP and Iba-1 protein expression compared with control diet-treated non-ischemic control retina. However, CoQ_10_ treatment significantly decreased GFAP and Iba-1 protein expression in ischemic retina. Values are mean ± SD (*n* = 3 retinas/group). *^,*§*^Significant at *P* < 0.05 and **Significant at *P* < 0.01 compared with control diet-treated non-ischemic control retina or ^#^Significant at *P* < 0.05 and ^##^Significant at *P* < 0.01 compared with control diet-treated ischemic retina

Since CoQ_10_ is neuroprotective against retinal damage by high IOP-induced transient ischemic injury and other apoptotic insults [35, 36], we determined whether CoQ_10_ promotes RGC survival in ischemic retina using whole-mount immunohistochemistry for Brn3a, a marker for RGCs. Mean RGC density per retina for each group is presented in Supplementary Table 1. Non-ischemic control mouse retina had an average of 3329 ± 533 RGCs in the central, 3326 ± 446 RGCs in the middle and 2230 ± 420 RGCs in the peripheral areas (*n* = 7 retinas) (Fig. 3a, b; Supplementary Table 1). In comparison with non-ischemic control retina treated with control diet, control diet treatment showed about 32 % of RGC loss in ischemic retina (*P* < 0.01; Fig. 3a, b; Supplementary Table 1). In contrast, CoQ_10_ significantly promoted RGC survival by an approximate 21 % compared with control diet-treated ischemic retina (*P* < 0.05; Fig. 3a, b; Supplementary Table 1). Astroglial and/or microglial activation coincide with RGC degeneration in the hypertensive retina of human, rat or mouse (3, 37–39). To investigate whether CoQ_10_ blocks the activation of astroglial and microglial cells in ischemic retina, we performed whole-mount immunohistochemistry or Western blot analyses using antibodies raised against glial fibrillary acidic protein (GFAP), a marker for astroglial cells, or Iba1, a marker for microglial cells. We found that GFAP and Iba1 immunoreactivity were increased in activated astroglia and microglial cells in the GCL and nerve fiber layer of control diet-treated ischemic retina at 12 h, respectively (Fig. 4a). Further, astroglial and microglial activation are accompanied by RGC loss as indicated by significantly increased GFAP and Iba-1 protein expression by 2.89 ± 0.49- and 11.8 ± 2.59-fold in control diet-treated ischemic retina at 12 h, respectively (*P* < 0.05 for GFAP and *P* < 0.01 for Iba-1; Fig. 4b). In contrast, CoQ_10_ significantly decreased GFAP and Iba-1 protein expression by 2.00 ± 0.23- and 1.38 ± 0.77-fold in ischemic retina at 12 h compared with control diet-treated ischemic retina (*P* < 0.05 for GFAP and *P* < 0.01 for Iba-1; Fig. 4b), indicating that CoQ_10_ prevents activation of astroglial and microglial cells in ischemic retina by blocking oxidative stress. In addition, there were no significant changes in RGC survival and GFAP protein expression between control- and CoQ_10_-treated control mice (Supplementary Fig. 1 and Supplementary Table 2).

### CoQ_10_ blocks apoptotic cell death by decreasing Bax or by increasing pBad protein expression in ischemic retina

**Fig. 5:**
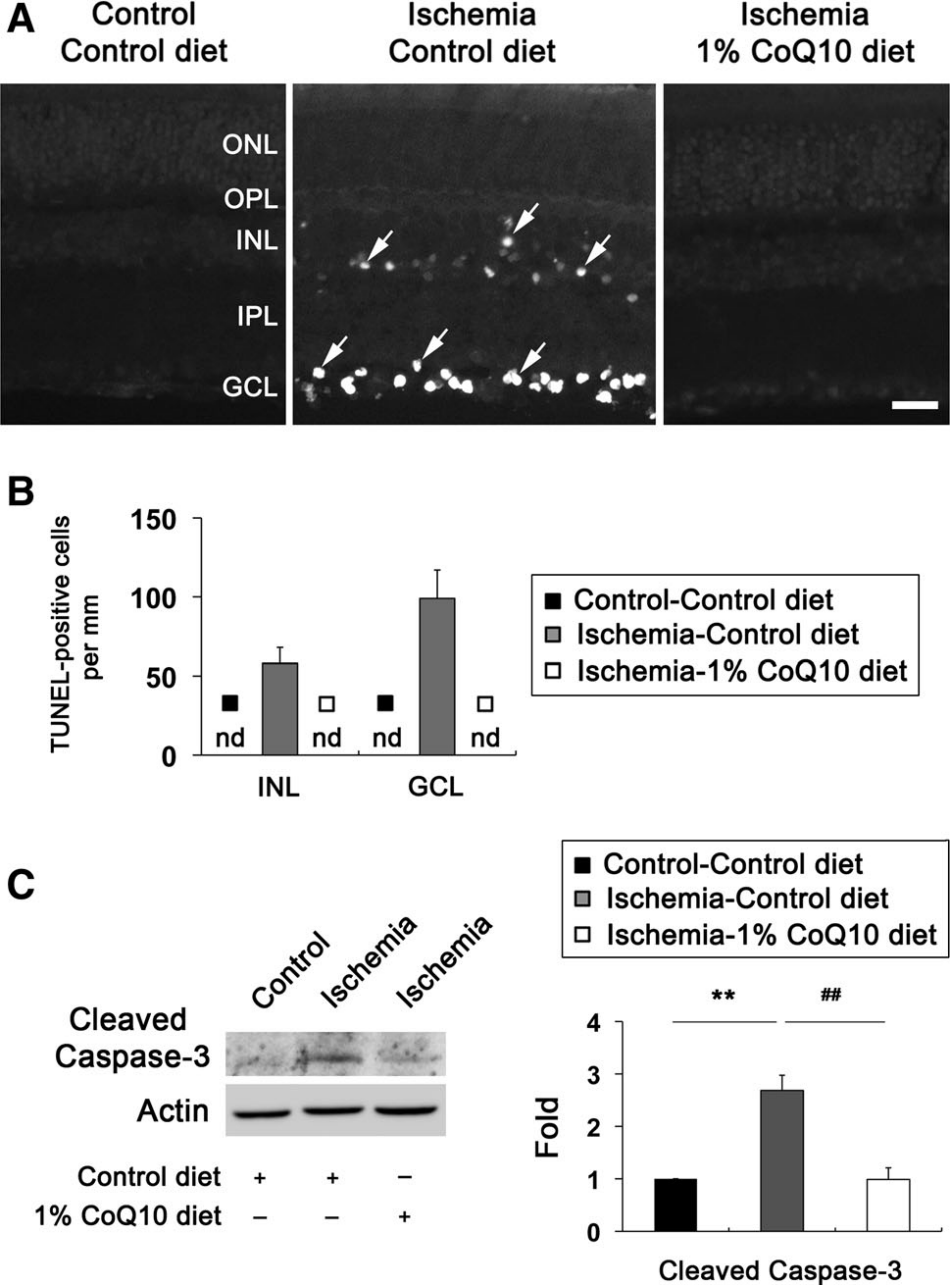
CoQ_10_-mediated blockade of apoptotic cell death in ischemic retina. Unsupplemented control or CoQ_10_ (1 %) diet daily were daily treated for 1 week before the induction of transient retinal ischemia and continued for 12 h.** a** TUNEL staining at 12 h after transient retinal ischemia. There were no TUNEL-positive cells in the GCL and INL of control diet-treated non-ischemic retina. However, control diet-treated ischemic retina showed TUNEL-positive apoptotic cell death in the GCL and INL at 12 h (*arrows*). In contrast, there were no TUNEL-positive cells in CoQ_10_ diet-treated ischemic retina. *INL* inner nuclear layer*, IPL* inner plexiform layer*, GCL* ganglion cell layer, *nd* non-detectable, *ONL* outer nuclear layer*, OPL* outer plexiform layer*. Scale bar* 20 μm.** b** Quantitative analysis of TUNEL-positive cells at 12 h after transient retinal ischemia. Values are mean ± SD (*n* = 5 retinas/group).** c** Caspase-3 Western blot analysis at 12 h after transient retinal ischemia. Control diet-treated ischemic retina significantly increased induction of cleaved caspase-3 protein expression compared with control diet-treated non-ischemic control retina. In contrast, CoQ_10_ treatment significantly decreased cleaved caspase-3 protein expression in ischemic retina compared with control diet-treated ischemic retina. Values are mean ± SD (*n* = 3 retinas/group). **Significant at *P* < 0.01 compared with control diet-treated non-ischemic control retina or ^##^Significant at *P* < 0.01 compared with control diet-treated ischemic retina

To determine whether CoQ_10_ prevents apoptotic cell death in ischemic retina, we performed TUNEL staining. There were no TUNEL-positive cells in the retina of control mice treated control diet (Fig. 5a, b). However, ischemic retinas showed TUNEL-positive apoptotic cell death in the inner nuclear layer (58 ± 10 per mm) and GCL (99 ± 18 per mm) at 12 h (Fig. 5a, b). In contrast, there were no TUNEL-positive cells in ischemic retina treated CoQ_10_ diet (Fig. 5a, b). In comparison with control diet-treated non-ischemic control retina, control diet-treated ischemic retina showed a significant induction of cleaved caspase-3 protein expression at 12 h by 2.68 ± 0.29-fold (*P* < 0.01; Fig. 5c). However, CoQ_10_ significantly reduced cleaved caspase-3 protein expression at 12 h by 0.98 ± 0.21-fold in ischemic retina (*P* < 0.01; Fig. 5c).

**Fig. 6:**
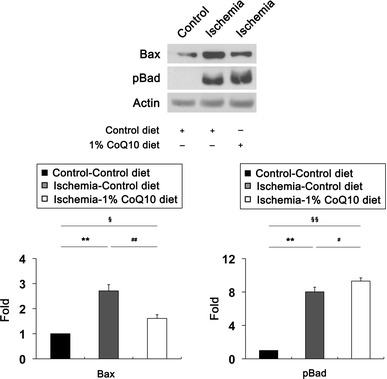
CoQ_10_-mediated blockade of apoptotic pathway in ischemic retina. Unsupplemented control or CoQ_10_ (1 %) diet were daily treated for 1 week before the induction of transient retinal ischemia and continued for 12 h. Bax and pBad Western blot analyses at 12 h after transient retinal ischemia. Control diet-treated ischemic retina significantly increased Bax and pBad protein expression. In contrast, CoQ_10_ treatment significantly decreased Bax protein expression, but increased pBad protein expression in ischemic retina compared with control diet-treated ischemic retina. Values are mean ± SD (*n* = 3 retinas/group). ^*§*^Significant at *P* < 0.05 and **^,*§§*^Significant at *P* < 0.01 compared with control diet-treated non-ischemic control retina or ^#^Significant at *P* < 0.05 and ^##^Significant at *P* < 0.01 compared with control diet-treated ischemic retina. *pBad* phosphorylated Bad

Our previous study showed that ischemic injury induces a significant increase of Bax and pBad protein expression in the early apoptotic pathway-mediated neurodegeneration of rat retina [5]. To determine whether CoQ_10_ modulates apoptotic cell death pathway in ischemic retina, we performed Western blot analysis using antibodies for Bax and pBad. We found that Bax protein expression was significantly increased by 2.71 ± 0.25-fold in ischemic retina at 12 h compared with control diet-treated non-ischemic control retina (*P* < 0.01; Fig. 6). In contrast, CoQ_10_ significantly decreased Bax protein expression by 1.61 ± 0.15-fold in ischemic retina at 12 h compared with control diet-treated ischemic retina (*P* < 0.01; Fig. 6). pBad protein expression was significantly increased by 8.02 ± 0.56-fold in ischemic retina at 12 h compared with control diet-treated non-ischemic control retina (*P* < 0.01; Fig. 6). Intriguingly, CoQ_10_ showed greater increase of pBad protein expression by 9.31 ± 0.39-fold in ischemic retina at 12 h compared with control diet-treated ischemic retina (*P* < 0.05; Fig. 6), suggesting that CoQ_10_ protects RGCs against oxidative stress-mediated apoptotic cell death by decreasing Bax or by increasing pBad protein expression in ischemic retina.

### CoQ_10_ preserves Tfam protein expression in ischemic retina

**Fig. 7:**
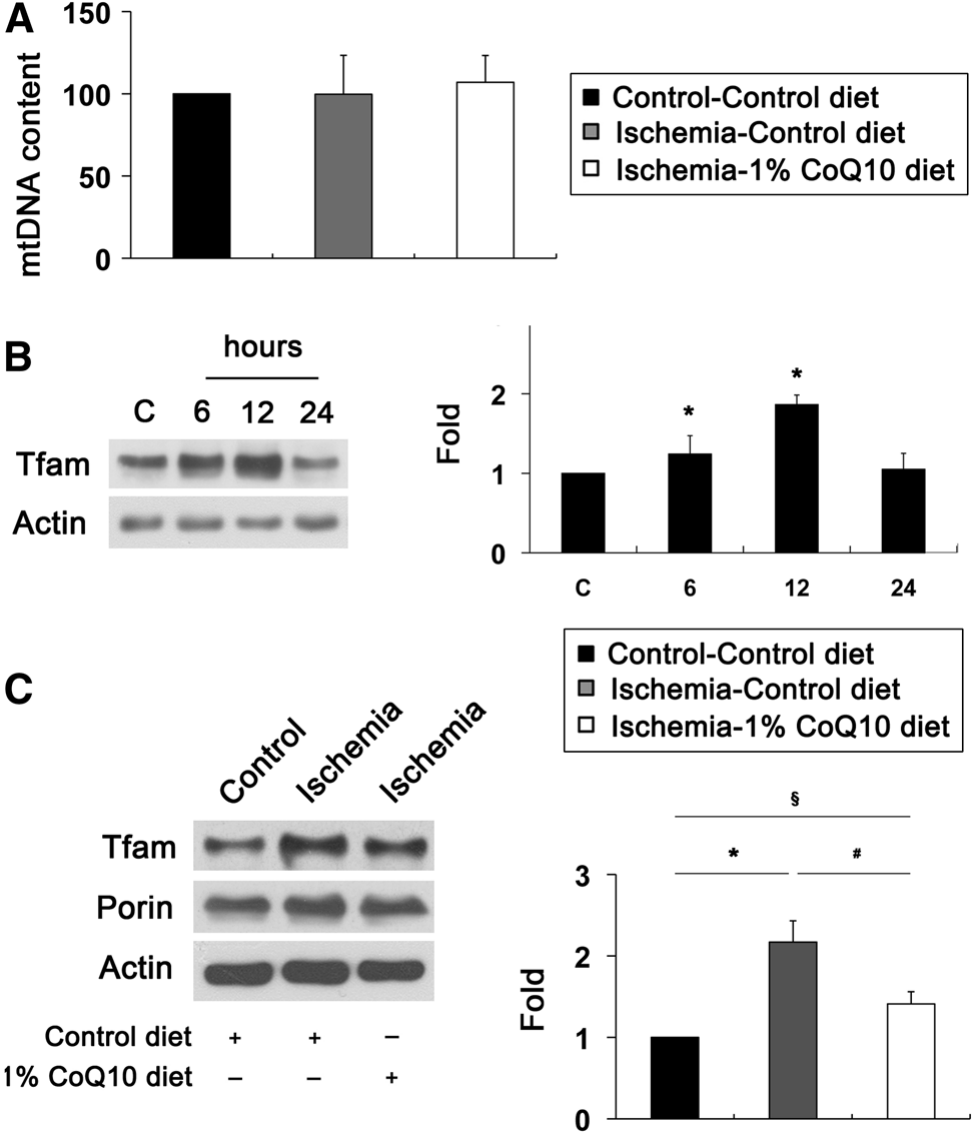
CoQ_10_-mediated restoration of Tfam protein expression in ischemic retina. Unsupplemented control or CoQ_10_ (1 %) diet were daily treated for 1 week before the induction of transient retinal ischemia and continued for 12 h. **a** mtDNA content at 12 h after transient retinal ischemia. Real-time PCR analysis showed that there were no differences in mtDNA content among groups. (b, and c) Tfam Western blot analysis at 12 h after transient retinal ischemia. **b** Tfam Western blot analyses at 6, 12 and 24 h after transient retinal ischemia. Increase of Tfam protein expression was maximal at 12 h in control diet-treated ischemic retina. The relative concentration of Tfam protein expression was less at 24 h than at 12 h after ischemia–reperfusion. **c** Tfam Western blot analysis at 12 h after transient retinal ischemia. CoQ_10_ treatment preserved Tfam protein expression at 12 h in ischemic retina compared with control diet-treated ischemic retina. Values are mean ± SD (*n* = 3 retinas/group). *^,*§*^Significant at *P* < 0.05 compared with control diet-treated non-ischemic control retina or ^#^Significant at *P* < 0.05 compared with control diet-treated ischemic retina. *Tfam* mitochondrial transcription factor A*, mtDNA* mitochondrial DNA

To determine whether oxidative stress induced by acute IOP elevation alters mtDNA content and whether CoQ_10_ preserves this alteration in ischemic retina, mtDNA content was determined by real-time PCR analysis. We found that there were no differences in mtDNA content among control diet-treated non-ischemic control, and control diet-treated ischemic and CoQ_10_-treated ischemic retinas at 12 h (Fig. 7a). Oxidative stress triggers increase of Tfam protein expression in ischemic rat retina [5]. To determine whether CoQ_10_ preserves this alteration in ischemic retina, we examined the expression level of Tfam using Western blot analysis. We observed that Increase of Tfam protein expression was maximal 12 h later by 1.86 ± 0.12-fold in ischemic retina (*P* < 0.05; Fig. 7b). The relative expression level of Tfam protein was less at 24 h than at 12 h in ischemic retina. Intriguingly, CoQ_10_ treatment partially preserved Tfam protein expression at 12 h compared with control diet-treated ischemic retina (*P* < 0.05; Fig. 7c). However, there was no difference in expression level of porin protein between control diet- and CoQ_10_ diet-treated ischemic retina. These results suggest that oxidative stress induced by acute IOP elevation increases Tfam protein expression but shows no alteration of mtDNA content in ischemic retina.

## Discussion

CoQ_10_ is an attractive neurotherapeutic agent for retinal protection against high IOP elevation-induced oxidative stress in retinal ischemic injury [24, 25] as well as other neurodegenerative diseases including Parkinson’ and Huntington’s diseases [20, 26, 40–42]. Moreover, CoQ_10_ is neuroprotective in retinal cells in vivo and in vitro against oxidative stress and excitotoxicity [23, 35, 41]. It has been reported that that CoQ_10_ supplements correlated with plasma CoQ_10_ level and CoQ_10_ in large doses was taken up all tissues including heart and brain mitochondria [43], suggesting that CoQ_10_ could also be taken up by retina and lead to a beneficial effect in ischemic retina injury. In support of this notion, a recent study demonstrated that oral supplementation of CoQ_10_ was neuroprotective against retinal damage induced by intravitreal injection of N-methyl-d-aspartate in mice in vivo [23]. In the current study, we addressed the question of whether a CoQ_10_-supplemented diet inhibits oxidative stress-mediated mitochondrial dysfunction and RGC degeneration in ischemic retina.

We previously reported that acute IOP elevation triggers the upregulation of SOD2 protein expression and this increase could be restored by brimonidine (BMD)-mediated blockade of glutamate excitotoxicity-induced oxidative stress in ischemic rat retina [5]. Consistent with these results, we have now found that the upregulation of SOD2 and HO-1 protein expression by acute IOP elevation was maximal early the neurodegeneration (12 h) of retinal ischemia. However, CoQ_10_ treatment decreases SOD2 and HO-1 protein expression in ischemic retina. Endogenous antioxidants such as mitochondrial SOD2 that localizes in the mitochondrial matrix and catalyzes the dismutation of superoxide radicals normally regulate ROS generated in mitochondria [44, 45]. It has been reported that SOD2 expression is increased by the stimulation of mitochondrial superoxide anion (O_2_^−^) [46] and CoQ_10_ treatment could reduce the O_2_^−^ production [47]. Growing evidence indicates that overexpression of the *SOD2* gene inhibits retinal damage by reducing oxidative stress and nitrosative stress [48], as well as protecting retinal vascular cells and capillary degeneration against apoptotic cell death in mouse models of ischemic injury [49]. Moreover, the upregulation of SOD2 is accompanied by a higher resistance to oxidative stress as well as SOD2 activity directly stabilizing mitochondrial transmembrane potential and calcium buffering ability [50, 51]. Our results, therefore, raise the possibility that increasing SOD2 or HO-1 expression in retinal ischemic injury may contribute to compensatory endogenous antioxidant mechanisms that increase resistance or stabilization of mitochondria against acute IOP elevation-induced oxidative stress. Taken together, it is noteworthy that these results suggest that CoQ_10_ could be an important antioxidant for ameliorating oxidative stress-mediated neurodegeneration in ischemic retina. Studying the benefits of CoQ_10_-mediated blockade of oxidative stress or enhancement of antioxidant enzymes may be a novel strategy for protecting retinal neurons against ischemic injury.

In the current study, we observed that CoQ_10_ treatment significantly promoted RGC survival as well as blocked activation of astroglial and microglial cells in ischemic retina. Astroglial or microglial activation coincides with RGC degeneration in the hypertensive retina of human, rat or mouse [3, 5, 37–39]. Thus, it is possible that CoQ_10_-mediated blockade of oxidative stress may indirectly reduce astroglial or microglial activation in the retina against elevated IOP-induced ischemic damage as a result of increased RGC survival. Regardless, it cannot be excluded that CoQ_10_ may prevent glial activation by blocking oxidative stress in astroglial or microglial cells in ischemic retina. CoQ_10_ is also neuroprotective in retinal glial cells against oxidative stress, since oxidative stress has remarkably linked to glial cell activation or reaction in the retina or optic nerve still needs to be ascertained [5, 52–56]. In addition, interestingly, it has been reported that IOP induced the upregulation of GFAP and major histochompatibility complex class II molecule, as well as of microglial activity in the controlateral control retinas in experimental rodent models of glaucoma induced by laser treatment [57, 58]. Although there was no evidence of glial activation in the contralateral non-ischemic control retina in the current study, future studies will need to clarify to use contralateral retina as an internal control in our ischemic model of mice.

Consistent with our result that CoQ_10_ promoted RGC survival in ischemic retina, we found that CoQ_10_ significantly decreased Bax, but increased pBad protein expression in ischemic retina. Bax is a pro-apoptotic member of the Bcl-2 family that is essential in many apoptotic pathways [59, 60] as well as directly interacts with the component forming the mitochondrial permeability transition pore (MPTP) that allows proteins to escape from the mitochondria into the cytosol to initiate apoptosis [61–63]. Bax is counteracted by Bcl-xL that forms heterodimers with dephosphorylation of Bad, which inactivates Bcl-xL; and pBad eliminates this dimerization, which activates Bcl-xL [64, 65]. Our collective results suggest that CoQ_10_ promotes RGC survival against the mitochondria-related apoptotic pathway in ischemic retinal injury by decreasing Bax, but by increasing pBad protein expression. These results reflect the possibility that increased pBad expression may represent an endogenous repair mechanism against apoptotic pathway and that CoQ_10_ may contribute to the blockade of Bax-mediated increase of MPT or the promotion of mitochondrial homeostasis in ischemic injury. The MPTP opening leads to loss of mitochondrial membrane potential, mitochondrial swelling, rupture of the outer mitochondrial membrane and release of cytochrome c. Importantly, MPTP opening by calcium overload and oxidative stress leads to necrosis rather than apoptosis [66, 67]. Recently, MPTP-mediated neuronal cell death has been implicated as an important pathophysiological mechanism for mitochondrial dysfunction in ischemic injury [24, 25]. Taken together, out findings suggest that CoQ_10_ may promote RGC survival by inhibiting not only apoptosis but also necrosis in ischemic retinal injury.

Ischemic injury triggers mitochondrial dysfunction including ROS induction as well as mtDNA damage, Tfam alteration or OXPHOS impairment in the central nervous system [5, 19, 68]. Recently, we have reported that acute IOP elevation significantly increased Tfam/OXPHOS complex protein expression and that blockade of glutamate excitotoxicity-induced oxidative stress by BMD treatment preserved Tfam/OXPHOS complex protein expression in ischemic retina [5], suggesting that the preservation of Tfam/OXPHOS complex may provide therapeutic potential for protecting RGCs against mitochondrial dysfunction induced by glutamate excitotoxicity and/or oxidative stress in ischemic retina [5]. In the current study, we observed that retinal ischemic injury triggers significant increase of Tfam protein expression in the early neurodegenerative events (6–12 h) and CoQ_10_ decreases Tfam protein expression in ischemic retina at 12 h. Emerging evidence suggests that the Tfam and OXPHOS complex IV are rapidly increased in the early neurodegenerative events of neonatal hypoxic-ischemic brain injury, suggesting that Tfam may contribute to endogenous repair mechanism of injured brain neurons [19]. Further, recent studies reported that overexpression of Tfam protects mitochondria against β–amyloid-induced oxidative damage in human SH-SY5Y neuroblastoma cells and ameliorates delayed neuronal cell death in the hippocampus following transient forebrain ischemia in mice [16, 17]. In contrast, in mice lacking *Tfam* there is impaired mtDNA transcription and mtDNA loss is triggered; this leads to mitochondrial bioenergetic dysfunction-mediated embryonic lethality [12].

Based on these results, we suggest that CoQ_10_-mediated preservation of Tfam expression may provide a potential mechanism for protecting RGCs against oxidative stress-induced mitochondrial dysfunction in ischemic retina. Future studies will examine the precise molecular mechanism underlying CoQ_10_-mediated Tfam preservation in ischemic retina. Although mtDNA is particularly susceptible to oxidative stress [69], it is unknown whether oxidative stress can directly trigger mtDNA alteration in ischemic retina. Interestingly, we found that there was no statistical difference in mtDNA content among control diet- and CoQ_10_ diet-treated groups. Because it has been reported that Tfam regulates mtDNA copy numbers in mammals and the levels of Tfam correlate with the levels of mtDNA [15, 70], our findings that did not show a correlation between Tfam protein expression and mtDNA content raise the possibility that increasing Tfam protein expression may prevent alteration of mtDNA content against oxidative stress in ischemic retina. Regardless, it would be useful to investigate mtDNA alteration on ischemic retina using measurements for 8-hydroxy-2-deoxyguanosine and mitochondrial membrane potential, and future studies need to address this more detail.

In conclusion, these results provide direct evidence that CoQ_10_ promotes RGC survival in ischemic mouse retina by inhibiting oxidative stress and by blocking the Bax/Bad-mediated mitochondrial apoptotic pathway. Moreover, CoQ_10_ also preserves Tfam protein expression in ischemic mouse retina. Based on these observations, we suggest that CoQ_10_ may provide a promising therapeutic potential for ameliorating oxidative stress-mediated mitochondrial dysfunction in ischemic retinal injury.

## Electronic supplementary material

Below is the link to the electronic supplementary material.

Supplementary material 1 (DOCX 552 kb)

Supplementary material 2 (DOCX 68 kb)

Supplementary material 3 (DOCX 56 kb)
