# Supplementary material for: Coenzyme Q10 ameliorates oxidative stress and prevents mitochondrial alteration in ischemic retinal injury
Source: Apoptosis. 2013 Dec 12;19(4):603–14. doi: 10.1007/s10495-013-0956-x (PMC3938850; doi:10.1007/s10495-013-0956-x)
Supplement: Supplementary file 1 — Supplementary material 1 (DOCX 552 kb) [file 10495_2013_956_MOESM1_ESM.docx]

Supplementary Fig. 1

**
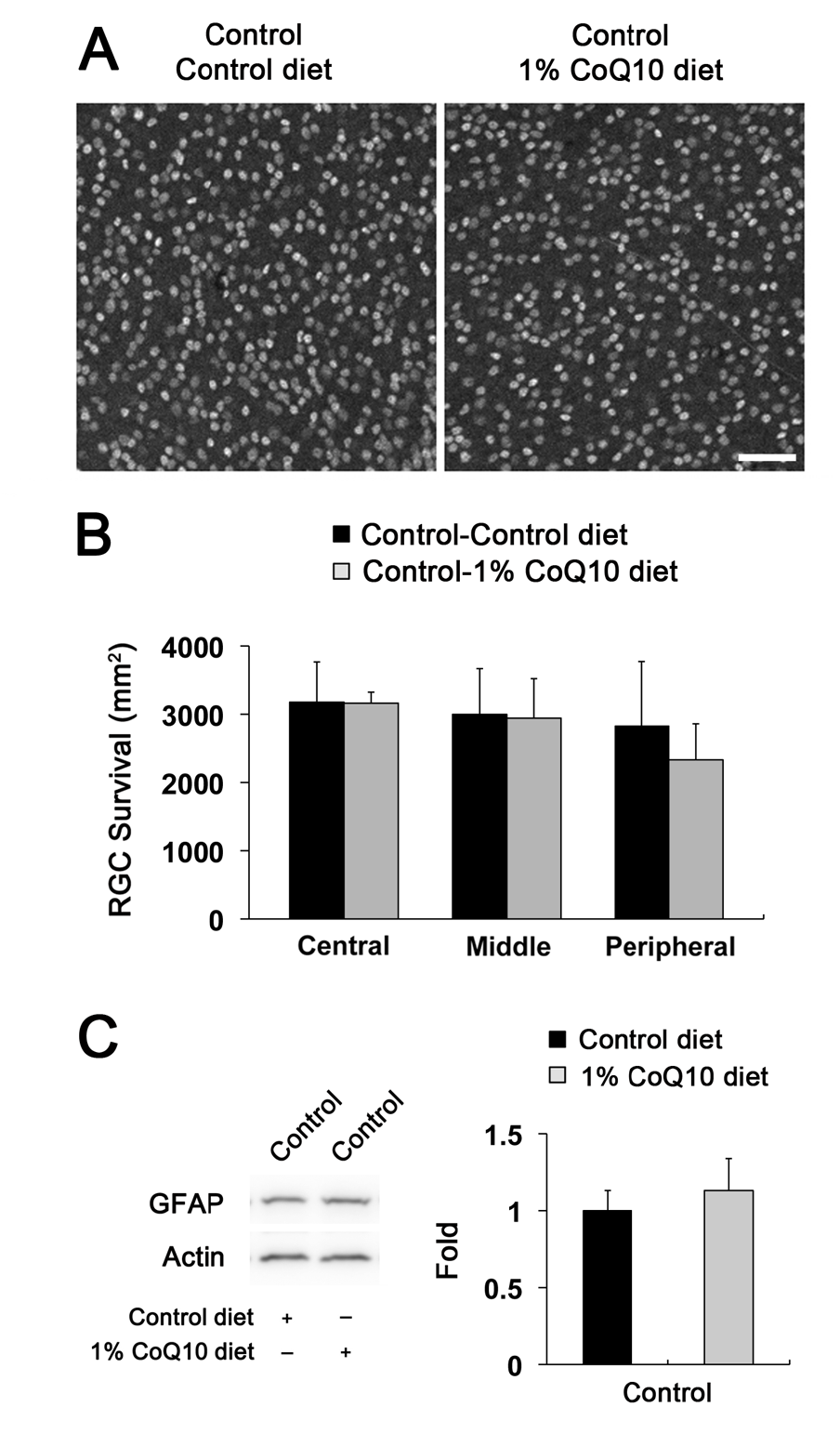
**

**Supplementary Fig. 1** RGC survival and GFAP protein expression in control mice treated with control or CoQ_10_ diet. Unsupplemented control or CoQ_10_ (1%) diet were daily treated and continued for 2 weeks. (A) Retinal whole-mount immunohistochemistry for Brn3a at 2 weeks after transient retinal ischemia. High magnification showed representative images from the middle area of retinas. (B) There were no significant changes in RGC survival between control- and CoQ_10_-treated control C57BL mice (*n* = 5 retinas/group). *Scale bar* 50 μm. (C) GFAP Western blot at 12 h after transient ischemia. There was no significant change in GFAP protein expression between control- and CoQ_10_-treated control mice.
