# Supplementary material for: Coenzyme Q10 ameliorates oxidative stress and prevents mitochondrial alteration in ischemic retinal injury
Source: Apoptosis. 2013 Dec 12;19(4):603–14. doi: 10.1007/s10495-013-0956-x (PMC3938850; doi:10.1007/s10495-013-0956-x)
Supplement: Supplementary file 2 — Supplementary material 2 (DOCX 68 kb) [file 10495_2013_956_MOESM2_ESM.docx]

**Supplementary Table 1**. Effect of CoQ_10_ on the central, middle, and peripheral RGC survival in ischemic retina at 2 weeks.

| **RGC density per retina (RGCs/mm^2^)** | | | |
| --- | --- | --- | --- |
| Treatment | **Central** | **Middle** | **Peripheral** |
| Non-ischemic control/Control diet | 3329 ± 533 | 3326 ± 446 | 2230 ± 420 |
| Ischemia/Control diet | 2410 ± 325^**^ | 2134 ± 217^**^ | 1499 ± 246^**^ |
| Ischemia/1% CoQ_10_ diet | 2775 ± 209^#,^***^§^*** | 2610 ± 258^#,^***^§^*** | 1916 ± 217^#,^***^§^*** |

Data are expressed as the mean ± SD. Comparison of three experimental conditions was evaluated using the one-way analysis of variance and the Bonferroni *t*-test.

^**^Significant at *P* < 0.01 compared with non-ischemic control retina treated with control diet

^#^Significant at *P* < 0.05 compared with ischemic retina treated with control diet.

***^§^***Significant at *P* < 0.05 compared with non-ischemic control retina treated with control diet.
