# Supplementary material for: Coenzyme Q10 ameliorates oxidative stress and prevents mitochondrial alteration in ischemic retinal injury
Source: Apoptosis. 2013 Dec 12;19(4):603–14. doi: 10.1007/s10495-013-0956-x (PMC3938850; doi:10.1007/s10495-013-0956-x)
Supplement: Supplementary file 3 — Supplementary material 3 (DOCX 56 kb) [file 10495_2013_956_MOESM3_ESM.docx]

**Supplementary Table 2**. Effect of CoQ_10_ on the central, middle, and peripheral RGC survival at 2 weeks in non-ischemic control retina.

| **RGC density per retina (RGCs/mm^2^)** | | | |
| --- | --- | --- | --- |
| Treatment | **Central** | **Middle** | **Peripheral** |
| Non-ischemic control/Control diet | 3178 ± 588 | 2999 ± 670 | 2827 ± 945 |
| Non-Ischemic control/1% CoQ_10_ diet | 3163 ± 162 | 2943 ± 580 | 2332 ± 528 |

Data are expressed as the mean ± SD. Comparison of two groups was evaluated using the unpaired, two-tailed student’s *t*-test.

There was no statistical difference in RGC survival between control diet- and CoQ_10_ diet-treated non-ischemic control mice (*n* = 5 retinal flatmounts/group).
